# Therapeutic accuracy of the completely customized lingual appliance WIN: A retrospective cohort study

**DOI:** 10.1007/s00056-016-0058-9

**Published:** 2016-11-17

**Authors:** Alexander Pauls, Manuel Nienkemper, Rainer Schwestka-Polly, Dirk Wiechmann

**Affiliations:** 1 Kieferorthopädische Fachpraxis Baden-Baden, Sophienstraße 22, Im Kaiserhof, 76530 Baden-Baden, Germany; 2 Poliklinik für Kieferorthopädie, Universität Düsseldorf, Düsseldorf, Germany; 3 Klinik für Kieferorthopädie, Medizinische Hochschule Hannover, Hanover, Germany; 4 Kieferorthopädische Fachpraxis Bad Essen, Bad Essen, Germany; 5 Kieferorthopädische Fachpraxis Düsseldorf, Düsseldorf, Germany

**Keywords:** Lingual brackets, Completely customized lingual appliance, Therapeutic accuracy, Treatment planning, 3D scan, Lingualtechnik, Vollständig individuelle linguale Apparatur, Behandlungsgenauigkeit, Behandlungsplanung, 3-D Scan

## Abstract

**Objective:**

The aim of this retrospective cohort study was to assess the accuracy of the completely customized lingual appliance WIN (DW Lingual Systems, Bad Essen, Germany) employing a three-dimensional (3D) comparison between the setup and the final result.

**Materials and methods:**

The setup and final models of 20 consecutively debonded patients (40 jaws; 7 males, 13 females; mean age 15.76 ± 4.45 years) with various malocclusions of a private practice specialized in orthodontics were digitalized using a 3D scanner. The 3D models of the setup and the final model of each jaw were then digitally matched using the best fit algorithm and segmented into single teeth. After placing individual coordinate systems, the homologous teeth of the setup and the final model were matched to be able to calculate the exact deviations of all rotational and translational components. The *t* test for unpaired samples, Kruskal–Wallis tests, *U* tests, and ANOVA with Duncan post hoc test were applied statistically.

**Results:**

Regarding the incisors, the angle discrepancies between the setup and the final result appeared to be less than 3° (torque 2.96°; tip 2.04°; rotation 2.00°). The translations showed mean values less than 0.3 mm (mesiodistal 0.16 mm; buccolingual 0.15 mm; vertical 0.29 mm). Slightly higher values could be measured in the lateral segments regarding rotations (torque 5.18°; tip 3.10°; rotation 3.70°) as well as regarding translations (mesiodistal 0.26 mm; buccolingual 0.64 mm; vertical 0.36 mm).

**Conclusions:**

Using the completely customized lingual appliance WIN, it is possible to achieve the final result predicted by the setup with a high accuracy.

## Zusammenfassung

**Ziel:**

Ziel der Studie war die Verifizierung der Behandlungsgenauigkeit der vollständig individuellen lingualen Behandlungsapparatur WIN (DW Lingual Systems, Bad Essen) durch dreidimensionalen Vergleich von Setup und Endergebnis.

**Material und Methoden:**

Setups und Endmodelle von 20 konsekutiv entbänderten Patienten mit unterschiedlichen Malokklusionen (40 Kiefer; 7 männlich, 13 weiblich; Durchschnittsalter 15,76 ± 4,45 Jahre) einer kieferorthopädischen Fachpraxis wurden mit einem 3-D-Scanner digitalisiert. Anschließend wurden die jeweiligen Scans der Setup- und Endmodelle mittels Best-Fit-Methode digital überlagert und in einzelne Zähne segmentiert. Daraufhin erfolgte der Matching-Vorgang für jeden Zahn, um die genauen Abweichungen bezüglich aller Rotationen und Translationen zu errechnen. Die Statistik umfasste *t*-Tests für ungepaarte Stichproben, Kruskal–Wallis-Tests, *U*-Tests und ANOVA mit Duncan Post-hoc-Test.

**Ergebnisse:**

Die Abweichungen zwischen Setup und Endergebnis betrugen im Frontzahngebiet für die angulären Messungen unter 3° (Torque 2,96°; Tip 2,04°; Rotation 2,00°) und translatorisch unter 0,3 mm (Mesial-/Distalstand 0,16 mm; in/out 0,15 mm; Supra-/Infraposition 0,29 mm). Im Seitenzahngebiet ergaben die Messungen leicht höhere Abweichungen sowohl für die Rotationen (Torque 5,18°; Tip 3,10°; Rotation 3,70°) als auch bezüglich der Translationen (mesial/distal 0,26 mm; bukko-lingual 0,64 mm; vertikal 0,36 mm).

**Schlussfolgerungen:**

Bei der Behandlung mit der vollständig individuellen lingualen Apparatur WIN kann das durch das Setup prognostizierte Endergebnis mit hoher Genauigkeit umgesetzt werden.

## Introduction

Since the late 20th century, as a result of the rising demand on the part of patients for an esthetic alternative to conventional labial brackets [12], many orthodontists have developed their own lingual techniques and appliances [2, 6, 10, 12, 18]. In addition to advantages shown for patients, the risk of developing white spot lesions appears to be drastically reduced when using lingual compared to labial appliances [7, 8, 15, 17].

**Fig. 1:**
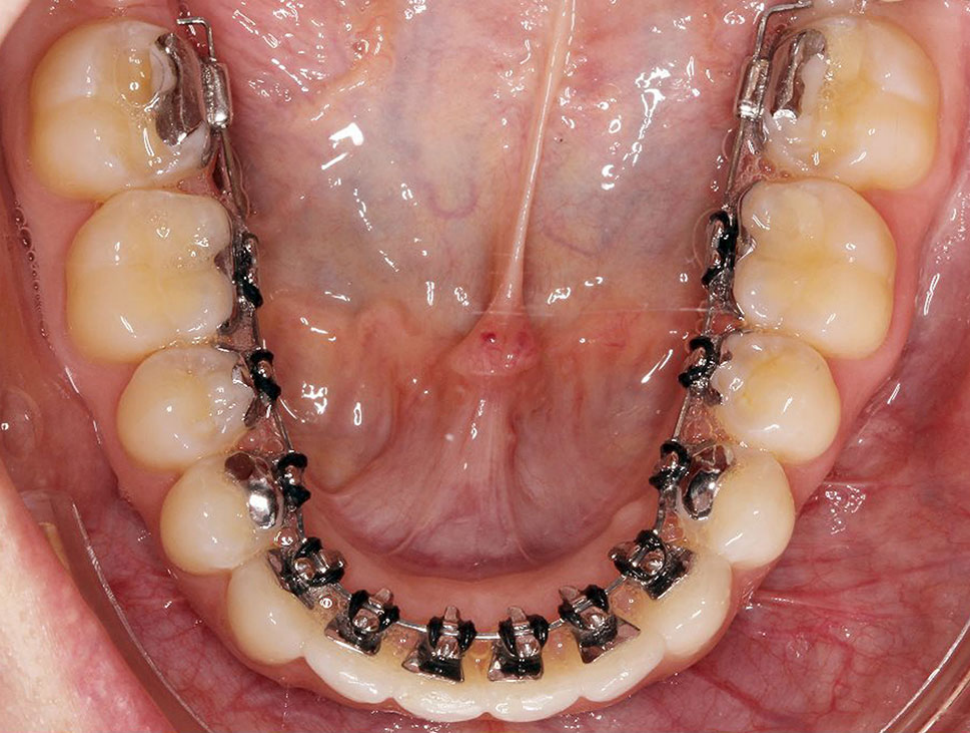
Intraoral picture of a lower jaw with the bonded, completely customized lingual appliance WIN after leveling and aligning **Abb. 1** Intraorales Foto eines Unterkiefers mit vollständig individueller lingualer Apparatur WIN nach der Nivellierungsphase

In order to produce the desired outcome in the patient’s dentition, not only the accuracy of brackets and archwires, the positioning and transfer system, but also the rebonding procedure have to be as good as possible. For the completely customized lingual appliance (CCLA) WIN (DW Lingual Systems, Bad Essen, Germany; Fig. 1), it has already been demonstrated that the accuracy of the slot dimensions [1], as well as that of the finishing archwires [9] is very high, providing good control of the desired tooth movements, including torque [9].

The use of digital models offers the possibility of combining different types of models (e.g., setup, malocclusion, and final result), as well as models made at different points in time using the same coordinate system [3, 4]. Numerous ways of comparing digital models three-dimensionally have been introduced [4].

Until now, no evaluation of the accuracy of the CCLA WIN has been made. The aim of the present study was to assess to which degree the completely customized lingual appliance WIN can realize the tooth positions planned by the setup using a three-dimensional (3D) analysis of all rotations and translations of each individual tooth.

## Materials and methods

### Materials

To compare the therapeutic accuracy of the CCLA WIN, in this retrospective study, the setup models, which were regularly made from a silicone impression of the malocclusion before beginning the treatment for the appliance’s production process, and final plaster casts made directly after removal of the appliance were compared three-dimensionally.

The study population comprised 40 jaws (20 upper, 20 lower) from 20 consecutively debonded patients (7 males, 13 females), whose mean age at the beginning of treatment was 15.76 ± 4.45 years. In 6 jaws of 4 patients, none of the second molars were erupted at the time of impression taking, resulting in a total of 548 examined teeth. At the beginning of the treatment 5 patients had an Angle Class I malocclusion (25%), whereas 13 patients presented an Angle Class II (65%), and 2 an Angle Class III (10%). No extractions were performed in any patient of the study group. The patients were included in this study by the first author without any further selection. The initial malocclusion was not a selection criterion. Patients with general diseases, clefts, or other syndromes affecting the cranial complex or bone remodeling, as well as cases in which surgical assistance was foreseen by the treatment plan were excluded. Patients with periodontal problems were also excluded. Steps were taken to ensure that no additional appliances had been used, such as Herbst and other fixed functional appliances, mini-implants, or extra-oral appliances, like headgear, so that it was possible to measure only the effect induced by the lingual bracket system itself. In most of the cases, elastics were utilized for finishing. In addition, only patients in whom no manual finishing bends by the clinician were necessary were chosen, thus, enabling us to analyze the tooth positions related to the appliance alone.

All patients were treated with the CCLA WIN and the same archwire sequence at the orthodontic practice of Prof. Dr. Wiechmann and partners in Bad Essen, Germany. The treatment was undertaken by different clinicians who all followed the same treatment procedures. In all patients, the treatment could be performed according to the treatment plan and none of the patients required their therapy to be discontinued. Treatment was commenced between June 2012 and June 2013. The debonding was undertaken between August 2014 and October 2014, resulting in a mean treatment time of 1.62 ± 0.30 years. If stripping was scheduled in the treatment plan, during setup manufacturing, the exact amount of stripping for each affected tooth surface was noted, and exactly transferred to the patient during treatment by the clinician. Neither the person who assessed the data nor the statistical expert were involved in the treatment of the patients. They both were blinded.

For the matching process of the teeth, it was essential that the plaster casts did not contain a bonded lingual retainer, buttons, or other attachments. In addition, patients were only included in the study in whom no restorations or fillings were carried out between commencement and end of treatment. Casts were inspected to exclude the possibility of them having any plaster beads or other flaws, such as broken or incomplete teeth.

No patients had to be excluded because of incomplete records. Four of the initial 24 patients had to be excluded because they did not meet all the inclusion criteria, resulting in a total of 20 patients being enrolled in the study. Two of these were excluded because a finishing bend was made by the practitioner, as the setup position of one particular tooth was not satisfactory. Since consecutively debonded patients were investigated, no selection was performed.

### Methods

The models were examined for plaster beads or other flaws which were then carefully removed if necessary. For each jaw, two models were scanned using a 3D scanner (Atos II Triple Scan, GOM, Braunschweig, Germany):

- The setup model was made during the production process of the completely customized lingual appliance WIN (setup model).
- The final plaster casts were made immediately after the debonding of the lingual appliance (final model).

The 3D scanner used is equipped with cameras with a resolution of 2 × 5,000,000 pixels and a point spacing of 0.02–0.79 mm (http://www.gom.com). The 3D data were saved in standard triangulation language (STL) format. These files were imported into the program Geomagic Studio 2014 (Version 2014.2.0.1765, Geomagic, Morrisville, NC, USA), together with a custom-made square plane and attached single coordinate systems for each tooth. Each of the 3D models was trimmed in a way that only the teeth were left—all gingival parts, as well as the alveolar crest, or possible scan artefacts, were removed. Then, the two models were segmented into individual teeth.

**Fig. 2:**
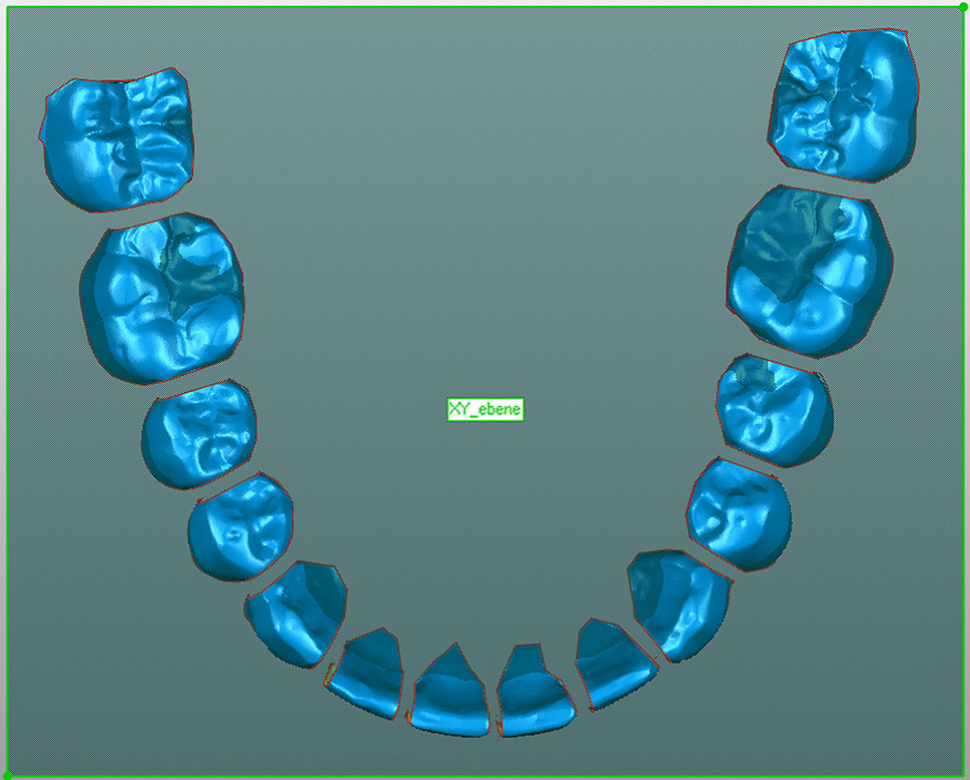
Lower jaw final 3D model after segmentation and alignment of the constructed plane (*greenish rectangle*); screenshot Geomagic Studio **Abb. 2** 3-D Abschlussmodell eines Unterkiefers nach Segmentierung und Ausrichtung der Hilfsebene (*grünes Rechteck*); Screenshot Geomagic Studio

**Fig. 3:**
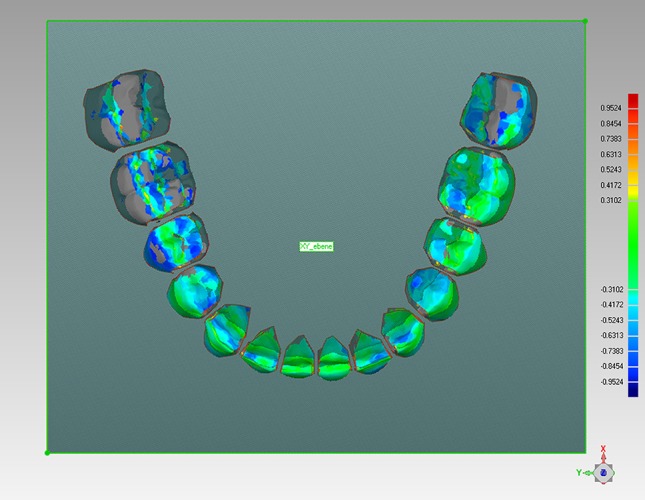
Lower jaw final model and setup model after the matching process. The accuracy of the matching process is visualized by the color scale on the *right side*; screenshot Geomagic Studio **Abb. 3** Abschluss- und Setupmodell eines Unterkiefers nach abgeschlossenem Matching-Prozess. Die Genauigkeit der Überlagerung wird durch die Farbskala am rechten Bildrand visualisiert; Screenshot Geomagic Studio

The final model was manually aligned to the plane in a manner such that the plane crossed every tooth at the approximate height of the bracket slots (Fig. 2) and was fixed in this position. Then, the setup model was moved over the final model manually in approximately the same position. Using the software’s global matching algorithm, the two models were then automatically matched, so that maximum compliance was ensured (Fig. 3). The two models were adjusted to the global coordinate system. Subsequently, the segmented individual teeth were renamed according to the tooth number. The segments of the final model were then fixed.

**Fig. 4:**
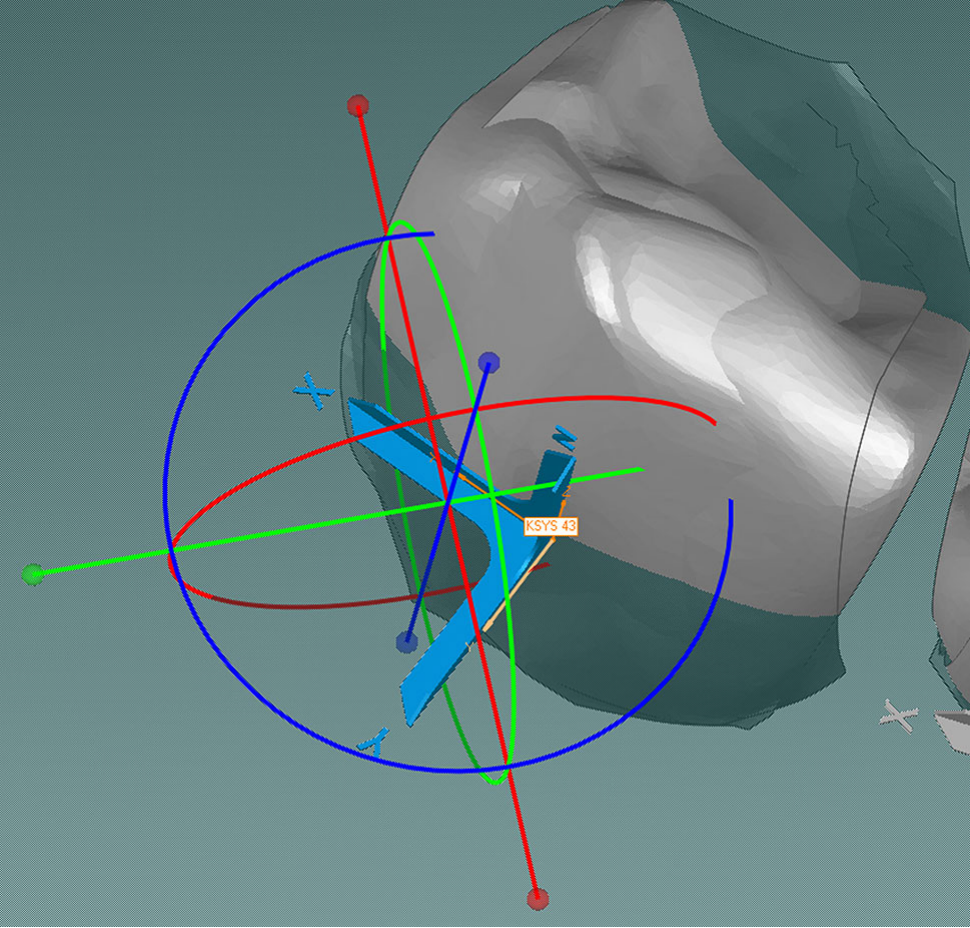
Detail view of tooth 23 during manual positioning of the coordinate system; screenshot Geomagic Studio **Abb. 4** Detailansicht des Zahnes 23 während der manuellen Positionierung des zugehörigen Koordinatensystems; Screenshot Geomagic Studio

The next step was to assign the coordinate systems to each tooth. To achieve most accurate results, all coordinate systems were located in a manner such that the *x*- and *y*-axis were positioned on the constructed square plane, with the *z*-axis perpendicular to it. The coordinate systems for the premolars and the molars were moved to the middle of the crown and, for the incisors and canines, the coordinate systems were positioned in the middle of the mesiodistal dimension of the crown’s buccal surface (Fig. 4). The coordinate system was positioned at the reference point of the setup tooth, as this was the tooth to be moved during the matching process. The single teeth of the setup were grouped together with their coordinate systems. Then, the coordinate systems were fixed and copied to the corresponding tooth of the final model, so that every tooth in both models had its own coordinate system.

**Fig. 5:**
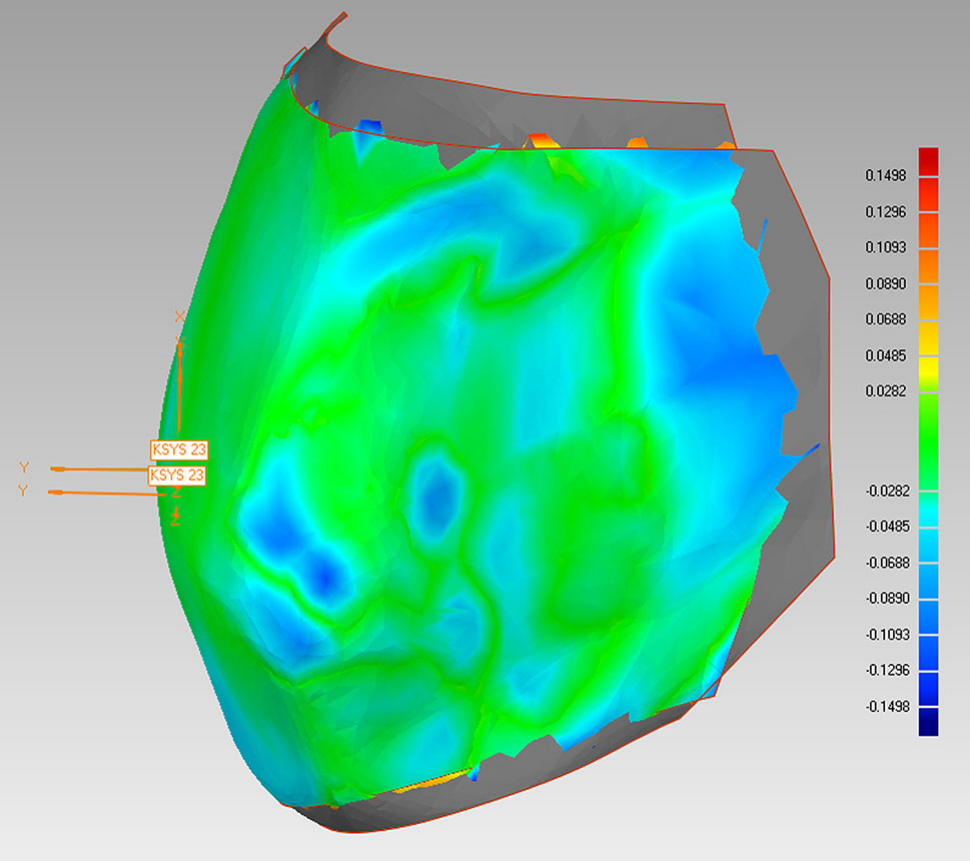
Detail view of tooth 23 after matching (final model and setup model); the accuracy of the matching process is visualized by the color scale on the *right side*. Average deviation for this particular tooth was 0.028 mm. The discrepancy of the two individual teeth is symbolized by the two *orange* coordinate systems; screenshot Geomagic Studio **Abb. 5** Detailansicht des Zahnes 23 nach dem Matching- Vorgang von Abschluss- und Setup-Modell; die Genauigkeit der Überlagerung wird durch die Farbskala am rechten Bildrand visualisiert. Die durchschnittliche Abweichung für diesen Zahn betrug 0,028 mm. Die abweichende Zahnstellung dieser beiden Zähne von Setup- und Abschlussmodell wird durch die 2 *orangen* Koordinatensysteme symbolisiert; Screenshot Geomagic Studio

For the matching process, homologous teeth, e.g., left central incisor of both the setup and the final model, were matched using the software’s automated best fit registration routine with the setup tooth being moved (Fig. 5). Consequently, the different positions of each tooth’s coordinate system could be the output for the translation of the *x*-, *y*-, and *z*-axis in millimeters (mesiodistal, vertical, buccolingual position), as well as for the rotation around the three axes, in degrees (rotation, tip, and torque).

The accuracy of the best fit method was automatically calculated by the software after each matching process and transferred to an Excel sheet.

If a corresponding pair of teeth could not be successfully matched automatically by the software, a manual *n*-point registration was carried out. Accordingly, three corresponding points of the two teeth were manually chosen. Using this information, the software registered the two teeth. Subsequently, the automated registration could always be successfully completed. The movements of the *n*-point registration were added by the software to those of the automated registration in order to calculate the full extent of the deviations of the teeth.

The variables for every of the 40 jaws were then transferred to an Excel sheet (Microsoft Excel 2010, version 14.0.7145.5000, Microsoft Corporation, Redmond, WA, USA).

This study has been approved by the ethics committee of the Hannover Medical School (No. 3151-2016).

### Reproducibility

Ten randomly selected jaws were re-examined 4 weeks after completion of the measurements by the same examiner to assess the reproducibility of the measurements and the accuracy of the method used. The double measurements revealed no single discrepancy. Because of the computed algorithm, for every single tooth, the best fit matching showed the exact same result as that measured at first. This result indicates the high level of reproducibility of the method used in this study.

### Statistical analysis

Statistical analysis was carried out using SPSS (Version 22, IBM, Armonk, NY, USA). All data were examined for normal distribution employing the Shapiro–Wilk test. When comparing upper versus lower jaw, a normal distribution was found. Therefore, the *t* test for unpaired samples was chosen for data analysis. For the evaluation of discrepancies between the different types of teeth (e.g., lower central incisors, upper first premolars), Kruskal–Wallis tests and *U* tests were applied, because of an absence of a normal distribution. The level of significance of the single *U* tests following a Bonferroni correction was *p* < 0.007. When comparing the different types of tooth movement, normal distributions were found. Therefore, statistical tests comprising an analysis of variance (ANOVA) with Duncan post hoc test were implemented.

The level of significance was set to *p* < 0.05, whereby *p* values <0.001 were regarded as being highly statistically significant.

## Results

The mean discrepancy of the matching of homologous teeth appeared to be 0.029 ± 0.009 mm. The mean values of the discrepancies between the setup and the final result are shown in Table 1 for each tooth. For torque, the second molars of the lower jaw showed by far the highest deviations; the upper right first molar and the lower right central incisor the smallest. When comparing the values for tip, the second molars showed the largest discrepancies, whereas tooth 14 showed the smallest. For the upper left molars, the differences between setup and the final result were the largest in relation to rotation and, for the teeth 11 and 22, the smallest. Regarding translations in the mesiodistal direction, the lower left incisors showed the smallest deviations; the highest were measured for all second molars. For the buccolingual dimension, the lower incisors were found to have the smallest discrepancies; the largest again were the second molars, with mean deviations of more than 1 mm. The second molars also showed the largest discrepancies in vertical translation; the upper left first premolar the smallest.

**Tab. 1:** Mean values and standard deviations of all 548 teeth examined in this study for every rotation and translation **Tab. 1** Mittelwerte und Standardabweichungen der Rotationen und Translationen aller im Rahmen dieser Studie untersuchten 548 Zähne

Tooth	Rotation (°)			Translation (mm)		
	Torque	Tip	Rotation	Mesiodistal	Buccolingual	Vertical
11	3.12 ± 2.37	1.72 ± 1.20	1.64 ± 1.08	0.19 ± 0.12	0.18 ± 0.14	0.38 ± 0.32
12	3.23 ± 2.11	2.11 ± 1.89	2.11 ± 1.72	0.27 ± 0.15	0.22 ± 0.19	0.33 ± 0.23
13	3.45 ± 2.78	3.75 ± 3.63	2.70 ± 1.85	0.30 ± 0.31	0.31 ± 0.36	0.37 ± 0.23
14	3.37 ± 2.09	1.30 ± 1.31	4.20 ± 3.40	0.31 ± 0.29	0.42 ± 0.34	0.26 ± 0.20
15	2.50 ± 1.80	2.68 ± 1.94	2.81 ± 2.75	0.28 ± 0.27	0.65 ± 0.60	0.26 ± 0.29
16	2.09 ± 1.47	2.35 ± 1.80	4.10 ± 4.61	0.28 ± 0.28	0.79 ± 0.79	0.31 ± 0.29
17	5.07 ± 3.36	5.88 ± 2.99	4.30 ± 2.99	0.36 ± 0.29	1.10 ± 1.10	0.62 ± 0.47
21	4.04 ± 2.40	1.79 ± 1.35	2.07 ± 1.74	0.17 ± 0.13	0.15 ± 0.12	0.41 ± 0.35
22	3.00 ± 2.38	2.30 ± 2.07	1.64 ± 1.46	0.15 ± 0.13	0.16 ± 0.13	0.32 ± 0.32
23	3.86 ± 2.64	3.63 ± 3.29	2.58 ± 1.90	0.20 ± 0.16	0.21 ± 0.20	0.33 ± 0.22
24	3.48 ± 2.77	2.44 ± 2.00	4.36 ± 2.60	0.21 ± 0.22	0.29 ± 0.21	0.14 ± 0.09
25	3.94 ± 2.21	2.07 ± 1.72	3.88 ± 3.01	0.17 ± 0.18	0.42 ± 0.40	0.22 ± 0.16
26	3.25 ± 2.01	2.10 ± 1.49	4.76 ± 4.17	0.25 ± 0.23	0.71 ± 0.69	0.17 ± 0.17
27	4.32 ± 3.29	5.71 ± 3.06	5.85 ± 3.76	0.40 ± 0.40	1.16 ± 1.12	0.73 ± 0.47
31	2.52 ± 1.81	1.95 ± 1.35	1.85 ± 1.29	0.12 ± 0.07	0.12 ± 0.12	0.18 ± 0.14
32	2.30 ± 1.73	2.42 ± 1.73	2.39 ± 1.23	0.12 ± 0.10	0.08 ± 0.06	0.23 ± 0.15
33	4.81 ± 2.61	3.50 ± 2.36	4.36 ± 3.03	0.16 ± 0.14	0.21 ± 0.17	0.36 ± 0.28
34	7.42 ± 4.08	3.14 ± 1.82	3.58 ± 2.39	0.14 ± 0.11	0.37 ± 0.28	0.21 ± 0.14
35	5.37 ± 3.49	2.86 ± 3.04	4.00 ± 3.38	0.23 ± 0.14	0.55 ± 0.51	0.45 ± 0.37
36	4.83 ± 2.69	1.72 ± 1.57	2.81 ± 3.03	0.26 ± 0.19	0.86 ± 0.73	0.43 ± 0.48
37	14.32 ± 6.22	4.16 ± 2.98	2.96 ± 2.72	0.33 ± 0.25	1.53 ± 1.19	0.50 ± 0.51
41	2.28 ± 2.21	1.77 ± 1.13	1.73 ± 1.41	0.13 ± 0.14	0.12 ± 0.10	0.17 ± 0.18
42	3.18 ± 2.64	2.27 ± 1.95	2.54 ± 1.94	0.16 ± 0.19	0.13 ± 0.09	0.29 ± 0.20
43	5.12 ± 2.67	2.59 ± 2.43	3.97 ± 2.18	0.23 ± 0.32	0.19 ± 0.22	0.42 ± 0.30
44	5.85 ± 2.55	3.52 ± 1.81	3.23 ± 2.60	0.22 ± 0.28	0.40 ± 0.39	0.24 ± 0.22
45	4.41 ± 2.46	2.65 ± 2.26	2.56 ± 1.99	0.28 ± 0.27	0.56 ± 0.48	0.44 ± 0.36
46	5.23 ± 3.45	2.34 ± 1.69	3.59 ± 2.20	0.30 ± 0.24	0.94 ± 0.81	0.45 ± 0.45
47	12.40 ± 7.21	4.91 ± 3.25	3.82 ± 2.59	0.41 ± 0.28	1.48 ± 1.04	0.54 ± 0.37

In every angle and translational measurement, the incisors presented fewer deviations than the lateral teeth, with mean values for the rotations between 2.00° (rotation) and 2.96° (torque) and for the translations between 0.16 mm (mesiodistal) and 0.29 mm (vertical). For the posterior teeth, the largest discrepancies were found for the torque and the buccolingual dimension (Table 2). Comparing all 548 teeth assessed in this study, the mean differences in rotations were less than 4.6° (torque 4.53 ± 3.98°; tip 2.79 ± 2.41°; rotation 3.20 ± 2.74°) and less than 0.5 mm for the translations (mesiodistal 0.23 ± 0.23 mm; buccolingual 0.49 ± 0.67 mm; vertical 0.34 ± 0.32 mm).

**Tab. 2:** Mean values and standard deviations of all incisors and all posterior teeth (canines to second molars) for every rotation and translation **Tab. 2** Mittelwerte und Standardabweichungen der Rotationen und Translationen aller Frontzähne und aller Seitenzähne (Eckzahn bis zweiter Molar)

Rotation (°)		Translation (mm)	
Incisors			
Torque	2.96 ± 2.24	Mesiodistal	0.16 ± 0.14
Tip	2.04 ± 1.60	Buccolingual	0.15 ± 0.13
Rotation	2.00 ± 1.51	Vertical	0.29 ± 0.26
Posterior teeth			
Torque	5.18 ± 4.34	Mesiodistal	0.26 ± 0.25
Tip	3.10 ± 2.61	Buccolingual	0.64 ± 0.74
Rotation	3.70 ± 2.98	Vertical	0.36 ± 0.35

Comparing the mean values for the discrepancies between setup and final result, the torque for every tooth was, on average, found to be rather more negative than in the setup. Considering rotation, there is more likely to have resulted a distorotation of the teeth in the final result compared to the setup, but, in terms of tip, there was no real tendency to either side. In most teeth, the final result buccolingually was found to be narrower than the setup, especially in the area of the molars. In the final result, the teeth were rather lower vertically (infraposition) than in the setup; for the mesiodistal translation, there was again no tendency to either side.

When comparing upper and lower jaw, highly significant differences were found for the parameter torque (*p* < 0.001) and significant differences for rotation (*p* = 0.021) and buccolingual position (*p* = 0.021) of the teeth (Table 3). Using analysis of variance, we were able to demonstrate that there were highly significant differences between the various types of tooth movement for rotations (*p* < 0.000) and for translations (*p* < 0.000). The Duncan post hoc test revealed significant differences between torque (rot *x*) and tip (rot *y*), as well as for torque and rotation (rot *z*). For translational movements, all types differed significantly (Table 4).

**Tab. 3:** Comparison of upper and lower jaw employing a *t* test for unpaired samples; level of significance *p* < 0.05 **Tab. 3** Vergleich der Ober- und Unterkiefer mittels *T*-Test für ungepaarte Stichproben; Signifikanzniveau *p* < 0,05

	Rotation (°)			Translation (mm)		
	Torque	Tip	Rotation	Mesiodistal	Buccolingual	Vertical
Mean value differences	−2.16	−0.61	0.21	0.03	−0.68	−0.01
*p* values	<0.001*	0.332	0.021*	0.438	0.021*	0.751

* Value considered significant

**Tab. 4:** Comparison of types of tooth movement employing Duncan post hoc test; mean values for groups in homogenous subsets; level of significance *p* < 0.05 **Tab. 4** Vergleich der unterschiedlichen Arten der Zahnbewegung; Mittelwerte für Gruppen in homogenen Teilmengen; Rotationen in Grad, Translationen in Millimetern; rot *x* = Torque, rot *y* = Tip, rot *z* = Rotation, trans *x* = Mesial-/Distalstand, trans *y* = in/out, trans *z* = Supra-/Infraposition; Signifikanzniveau *p* < 0,05

Rotation (°)			Translation (mm)			
Axis	Subset for Alpha = 0.05		Axis	Subset for Alpha = 0.05		
	1	2		1	2	3
rot *y*	2.7896		trans *x*	0.2333		
rot *z*	3.1991		trans *z*		0.3426	
rot *x*		4.5335	trans *y*			0.4938

*rot x* torque, *rot y* tip, *rot z* rotation, *trans x* mesiodistal*, trans y* buccolingual, *trans z* vertical translation

In addition, we examined whether there were significant differences between the various types of teeth. The central and lateral incisors, canines, first and second premolars, and first and second molars of the upper, as well as of the lower jaw, were investigated in terms of all rotations and translations. For the upper jaw, in relation to torque (*p* = 0.22) and translation in the mesiodistal dimension (*p* = 0.74), no significant differences were detected between the various tooth types. In the lower jaw, all rotations and translations were significantly different. The second molars differed significantly from all other teeth regarding tip in the upper jaw and torque in the lower jaw. The incisors showed no significant differences in any rotation or translation to each other.

## Discussion

### Methodology

The accuracy of a different lingual bracket system has already been evaluated using similar methodology [4, 11]. In order to receive precise information about every three-dimensional (3D) discrepancy, the plaster casts have to be free from any plaster beads, broken teeth, or other flaws before digitalization, as was ensured in our study. The accuracy of the 3D scanner used also plays a crucial part in the digitalization of the models. The scanner used produced scanned data of high quality. In addition, the matching algorithm has a large influence on the accuracy of results. The software used has already been established as fulfilling the required performance criteria in another study [4]. Double measurements were carried out to verify the practicability and reproducibility of the complex best fit algorithm and to exclude methodological errors in relation to the algorithm used. The fact that none of the duplicate measurements of the best fit matching of homologous teeth showed any discrepancy proves the high reproducibility of this algorithm. The reason for this very high degree of reproducibility may be that the software always precisely uses the same mathematical procedure to find a position where the maximum possible parts of the teeth fit best. The accuracy of the matching process is comparable to that reported in another study [11]. In that study, the accuracy was below 0.07 mm; in the present study, an average accuracy of 0.029 ± 0.009 mm was found. This could be due to the improvement in the matching algorithm which has been achieved in the past few years.

For the matching process, it is also important to remove all elements not belonging to the dental crowns before using the best fit algorithm. Therefore, the gingival parts were thoroughly removed and the teeth were segmented. We also made sure that no additional elements, such as bonded lingual retainers or buttons were present in the plaster casts before scanning was carried out. The small mean discrepancy in the matching process below 0.03 mm could be explained by tooth wear due to ingestion and possible bruxism, as well as by possible alterations in the surface which the lingual appliance was bonded to. When removing the composite from the bonding surface, there is the possibility of remnants or a slight removal of enamel. Therefore, not only the lingual surface was used for the matching process, but also the whole dental crown, in order to provide the largest possible surface for this process and to reduce the effect of possible surface alterations. In all patients evaluated in this study, no fillings or restorations were made or changed by the dentist throughout the treatment period.

It must be critically mentioned that the initial matching of the whole jaws could lead to small discrepancies because the differences are averaged by the algorithm. There are no stable structures in the upper or lower jaw that remain exactly the same before treatment, when the setup is made, and after treatment, when the final models are produced. In most cases—as can be seen in Fig. 3—most of the dental arch fitted very well and only some of the teeth showed higher discrepancies. This may lead to the conclusion that the best fit method is feasible. The possibility of averaged discrepancies during initial matching of the whole jaws was taken into consideration when analyzing the data. Therefore, in each jaw, tooth couples (e.g., central incisors, first premolars) were always compared in the statistical analyses.

Because the tooth axis cannot be identified exactly using a model of the teeth without information on root positions and root morphology, a plane was chosen in which the *x*- and *y*-axis of all coordinate systems were located. The plane was placed in a manner such that the dental crowns of all teeth were crossed at the approximate height of the bracket slots representing the level of force application. With regard to the rotations, each coordinate system was positioned manually onto the mesiodistal center of the crown of the incisors and canines, and onto the center of the crown of the premolars and molars. With regard to the position of the coordinate systems, the tooth of the setup was always chosen because this was the tooth to be moved later during the best fit matching process. It must be mentioned that the translational and angular discrepancies influence each other. The values calculated always indicate the differences in the position of the homologous teeth in the center of the chosen coordinate system. However, the resulting translations or rotations of any other point of the dental crown can be computed with the values if necessary. The direction of discrepancies, e.g., if the torque is more positive or more negative in the final situation compared to the setup, could be influenced by the initial position of the teeth in the malocclusion.

By investigating the data of consecutively debonded patients, there was no bias in relation to treatment complexity, treatment duration, and type of malocclusion. Certain additional appliances, such as the Herbst appliance or forms of treatment, such as a surgical correction of a malocclusion, were excluded, as were interventions by the orthodontist using manually made finishing bends. In addition, patients with periodontal diseases were excluded because of biomechanical differences caused by the different position of the center of resistance of the teeth. This should provide the opportunity for measuring only the effects induced by the completely customized lingual brackets and archwires. Interproximal reduction was not an exclusion criterion because the amount of reduction planned on the setup and in the dentition were the same. It should be mentioned that in some cases class II elastics were used which could have affected rotations or translations. These effects are supposed to be quite small because the elastics were only used on 0.016 × 0.024″ stainless steel or 0.018 × 0.018″ slot-filling β-titanium archwires with tight steel ligatures in place.

### Results

The largest translational discrepancies between the setup and the final situation occurred in the buccolingual position of the second molars. The mean values for each second molar were slightly more than 1 mm with the setup being mostly transversally wider than the final result. It was also possible to show that the further distal, the more transversal discrepancies occurred. Also, all other translational discrepancies were mostly higher for the second molars than for the other teeth. This result is in accord with previous studies [4, 11] and could be due to the fact that even stiff and slot-filling archwires do not have the potential to fully convert the transversal dimension in the posterior areas from the setup into the patient’s dentition. In all patients, the occlusal pads on the second molars were removed one appointment before debonding, so that the settling into an ideal intercuspation was not fully controlled by the appliance. With regard to the translation in the mesiodistal dimension, no significant differences were found between tooth types in the upper jaw. Also, in relation to torque, there were no significant differences in the upper jaw. The latter seems surprising because the 0.016 × 0.024″ stainless steel, as well as the 0.018 × 0.018″ β-titanium archwires, were vertically not slot-filling in the ribbon-wise slots with horizontal insertion of the premolars and molars. The torque discrepancies for the lower second molars were more than twice as great as that of the upper second molars. This cannot be explained by the dependency of torque on first and second order movements [13, 16] because the discrepancies of the latter were even smaller with the lower second molars than with the upper second molars. One reason could possibly be a higher initial misalignment of these teeth and an even greater effect of the reduced movement capacity at the end of the wire due to the large root surface of the molars.

We know from the literature that the completely customized lingual appliance WIN differs from other lingual appliances [1, 5, 9]. The high accuracy of the bracket slots and archwires results in a very good control of the desired tooth movements. This is supported by findings from the present study. It should be mentioned that the results of the studies evaluating the accuracy of lingual appliances differ regarding the positioning of the coordinate systems for each tooth leading to possible discrepancies in measurement. Pauls [11] positioned the coordinate systems in the mesiodistal center of the lingual bracket slot, whereas Grauer and Proffit [4] placed them at the cervical end of the crown on the estimated tooth axis. The procedure of placement of the coordinate systems in the present study was elaborated and discussed above. Comparing the results of the present study with the prior publications mentioned above, assessing the accuracy of a different lingual appliance [4, 11], the mean discrepancies of the incisors showed lower values for every single rotation and translation in the current study. For torque, a reduction in differences between 18 and 31% was measured. With particular regard to the mesiodistal and buccolingual translations, the mean differences were less than half in this study than has been reported elsewhere. When comparing all the teeth, the study of Grauer and Proffit [4] reported slightly lower differences for torque (4.21° vs. 4.53°), the other rotations, and all of the translations presented lower mean differences in the present study. Compared to the previous study of Pauls [11], the mean differences for every rotation and translation were reduced in this study. A previous publication [14] also produced results in accord with the present study.

These superior values could be explained by the various changes implemented in the WIN system, as well as by the high precision of bracket slots and archwire dimensions [1]. The greater mesiodistal dimension of the bracket slots of the WIN bracket compared to others could be the reason for the improved tip and rotational control. The torquing capacity of the WIN system has already been evaluated in vitro [9]. It was shown that the 0.018 × 0.018″ β-titanium finishing archwire, which was used for every jaw included in this study, presents between 0° and 2° of effective torque play, which must be exceeded in order to generate an effective torque moment. Only after twisting the archwire 2°–3° was a moment of 2 Nmm achieved that was needed for an effective torque correction [9]. These findings are in agreement with the present study, since this initial torque play is already included in the mean discrepancies of 2.96° for the anterior teeth, which show effective torque control.

Since this study only assessed whether the planned tooth movements could be achieved by the appliance, the amount of movement of each tooth is not relevant to make a statement in this regard and could be the subject of further studies.

Finally, it should be noted that a fixed appliance can only position teeth in the respective jaw. The coordination of the two jaws must be optimized by using additional features, such as elastics. Moving teeth with a multibracket appliance always results in a multi-indeterminate system of forces affecting each other, which may impose a need for a compliance-dependent finishing stage at the end of treatment. Because of the uncertainty of patient cooperation, it is not valid to examine occlusion in a study concerned with the accuracy of such an appliance.

## Conclusions

- It is possible to plan the final result of orthodontic treatment using a setup and to realize these tooth movements with a high degree of accuracy.
- The best fit algorithm used in this study seems to be a very accurate and highly reproducible method for matching homologous teeth.
